# The ‘problematisation’ of palliative care in hospital: an exploratory review of international palliative care policy in five countries

**DOI:** 10.1186/s12904-016-0137-0

**Published:** 2016-07-25

**Authors:** Jackie Robinson, Merryn Gott, Clare Gardiner, Christine Ingleton

**Affiliations:** 1 School of Nursing, University of Auckland, Auckland, New Zealand; 2 Auckland District Health Board, Auckland, New Zealand; 3 School of Nursing, University of Sheffield, Sheffield, UK

**Keywords:** Palliative care, Policy, Hospital

## Abstract

**Background:**

Government policy is a fundamental component of initiating change to improve the provision of palliative care at a national level. The World Health Organisation’s recognition of palliative care as a basic human right has seen many countries worldwide develop national policy in palliative and end of life care. There is increasing debate about what form comprehensive palliative care services should take, particularly in relation to the balance between acute and community based services. It is therefore timely to review how national policy positions the current and future role of the acute hospital in palliative care provision. The aim of this exploratory review is to identify the role envisaged for the acute hospital in palliative and end of life care provision in five countries with an ‘advanced’ level of integration.

**Method:**

Countries were identified using the Global Atlas of Palliative Care. Policies were accessed through internet searching of government websites between October and December 2014. Using a process of thematic analysis key themes related to palliative care in hospital were identified.

**Results:**

Policies from Switzerland, England, Singapore, Australia and Ireland were analysed for recurring themes. Three themes were identified: preferences for place of care and place of death outside the hospital setting, unnecessary or avoidable hospital admissions, and quality of care in hospital. No policy focused upon exploring how palliative care could be improved in the hospital setting or indeed what role the hospital may have in the provision of palliative care.

**Conclusions:**

Palliative care policy in five countries with ‘advanced’ levels of palliative care integration focuses on solving the ‘problems’ associated with hospital as a place of palliative care and death. No positive role for hospitals in palliative care provision is envisaged. Given the rapidly increasing population of people requiring palliative care, and emerging evidence that patients themselves report benefits of hospital admissions, this area requires further investigation. In particular, a co-design approach to policy development is needed to ensure that services match the needs and wants of patients and families.

## Background

A recommendation from the World Health Organisation to recognise palliative care as a basic human right by adopting a public health approach to service development [1], has seen many developed countries move towards developing national policy in palliative and end of life care [2]. Furthermore, in response to the demands of an ageing population and the impact of people living longer with chronic disease, governments have focused on developing health care policy which seeks to identify ways to meet the predicted increase in demand for palliative and end of life care services [3]. At the same time, practices in health and social care with older people and those with complex needs are increasingly under the spotlight [4]. However, the development of palliative care policy has occurred within the context of a global recession which has seen increasing pressure to reduce public health spending while seeking ways to increase service capacity to meet future demand.

Whilst palliative care has developed into an established specialty area of clinical practice [5], it is more than just a technical skill; rather it is predicated upon a specific philosophical approach to care. The ideology of a ‘good death’ [6] and the World Health Organisation’s definition of palliative care is said to encapsulates a philosophy of palliative care [7] which is in line with that which has informed the development of the modern hospice movement. This philosophy continues to inform and guide palliative care policy and practice. According to Clark [8] the philosophy of a ‘good death’ incorporates the following elements: pain free death, open acknowledgement of the imminence of death, an ‘aware’ death in which personal conflicts are resolved, death as personal growth, death according to personal preference and death at home, surrounded by family and friends. Drawing on this framework, achieving a natural death free from medical technology at home became a major focus during the early period of the modern hospice movement [9]. Some have suggested that this way of conceptualising a good death, particularly outside the acute hospital, set palliative care and hospice up in opposition of mainstream healthcare’ [6].

However, the way in which hospitals are used in palliative care has changed dramatically since the start of the modern hospice movement. Early definitions of palliative care were limited to those with ‘terminal cancer’ when life prolonging treatments had been exhausted [10]. However, it became apparent that those dying from non-cancer illnesses such as heart failure and chronic obstructive respiratory disease received little or no palliative care and died with significant unmet need [11, 12]. In 2002 the World Health Organisation [13] provided the impetus to move palliative care further upstream in the illness trajectory, thereby seeking integration with curative and rehabilitation therapies and shifting the focus beyond the final stages of life. In addition the diagnostic remit of palliative care expanded to include patients with a non-cancer diagnosis for whom prognosis might be many months or even years away and this has seen a change in the way palliative care is provided [5]. For example, life limiting illnesses such as chronic obstructive respiratory disease and heart failure are characterised by exacerbations of illness requiring hospitalisation during which death may occur [14]. Moreover, an increase in the use of hospital based technology in palliative care, much of which can only be offered in an acute hospital setting, is also impacting on the way in which hospitals are being used.

It has been suggested that government policy is a fundamental component of initiating change to improve the provision of palliative and end of life care [2]. With an increasing emphasis on the development of national policy it is therefore timely to explore how hospitals are positioned as settings for palliative care. Therefore, the aim of this exploratory study is to identify how the role of the hospital is envisaged within national policy on palliative and end of life care.

### Data sources

In 2014 the World Palliative Care Alliance and the World Health Organisation developed a Global Atlas of Palliative Care [2] (GAPC) which quantified the need for and availability of palliative care worldwide. At the time of the report 20 countries had attained the ‘advanced integration’ level of palliative care development indicating that palliative care was well integrated within mainstream health care providers and had substantial impact upon policy (see Table 1). It was from this group of countries that the policies included in this review were identified. Policy was defined as any government led document written with the aim to identify gaps and inequities in service delivery and provide recommendations for service development in order to improve palliative and end of life care.

**Table 1:** Countries with advanced integration of palliative care (adapted from the GAPC, 2014)

Advanced integration (*n* = 20)	**Australia**, **Austria**, Belgium, **Canada**, **France**, Germany, Hong Kong, Iceland, Ireland, Italy, Japan, **Norway**, Poland, Romania, Singapore, Sweden, **Switzerland**, Uganda, **United Kingdom**, United States of America

Those in bold are countries with government policy in palliative and end of life care

Policies were accessed through internet searching of government websites between October-December 2014. Whilst some countries such as Germany and Belgium had palliative care regulations or legislations in place, in order to be included in the review countries had to have a government-led national palliative care strategy or policy (see Table 1). Due to the cost of translation, those documents not available in English were excluded from the review. Therefore Sweden, Norway, France and Austria were excluded. Although referred to as a ‘strategy’ the Canadian [15] document was largely a report on the progress of community based workgroups implementing recommendations from a government report. For this reason it was subsequently excluded from the final analysis. The United States was not included in the review as they do not have a Federal based policy in palliative care. Therefore policy documents from United Kingdom [16], Australia [17], Switzerland [18], Ireland [19] and Singapore [20] were included in the review.

## Methods

**Fig. 1:**
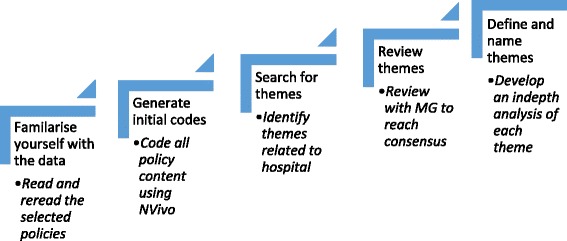
Methodology (adapted from Braun and Clark) [22]

An approach to thematic analysis as described by Braun and Clark [21] was used to explore policy content. This involved firstly familiarisation with the data through a process of reading and re-reading the policy documents, secondly a process of coding across the entire data set was completed using the software program *NVivo*. A general inductive approach was used to identify themes from those codes that were related to care and death in hospital. There was no predetermined coding frame; instead, this was developed as the data was coded. All coding was done by JR. The final steps in the analysis process involved the development of key themes which was achieved through a cyclical process of review and re-review of the relevant codes with consensus reached during regular meetings with MG. Finally an in-depth analysis of each key theme was undertaken (see Fig. 1).

## Results

Policies included in the review were published over a 10 year period with the earliest released in 2001 [19, 22] and the most recent in 2011 [20]. All policies adopted the WHO definition [13] as a framework to guide discussion. Furthermore, all used evidence from research to support the need to improve palliative care across multiple care settings, including the hospital. Policies acknowledged a need for palliative care to be integrated into mainstream health care. There was minimal evidence of consumer consultation in the development of policies with all countries appointing a combination of government employed policy analysts/makers, expert clinicians and leaders in palliative care to develop policy. A summary of the key points made in each policy document can be found in Table 2.

**Table 2:** Summary of key points

Country	Year	Authors	Summary of key points relating to hospital palliative care
Australia	2010	Policy makers	• People prefer to be cared for and die at home yet most die in hospital • Potential for cost savings by avoiding inappropriate hospital admissions • Burden of hospitalisation on the health care system and poor quality of death in hospital
England	2008	Advisory board supported by 6 key work groups	• People prefer to be cared for and die at home yet most die in hospital • Key strategy aim to reduce number of hospital deaths • Lack of community responsiveness results in admissions and prolonged hospital stay • Improved community provision reduces admissions enabling people to die in place of choice • Poor quality of care in hospitals • Care for dying people is a core role of the hospital in the ‘foreseeable future’
Ireland	2001	National advisory committee of clinicians, leaders and policy makers	• People prefer to be cared for and die at home yet most die in hospital • Unresponsive community services result in emergency hospital admissions • More investment in community services would reduce unnecessary hospital admissions • Poor quality of care in hospitals
Singapore	2011	Workgroup comprising of health professionals	• More understanding needed regarding people’s preferences at the end of life including preferences for place of care in Singapore • Majority of patients are admitted to hospital for symptom control and more patients are cared for in hospital than necessary • Home care teams need to be able to provide treatment at home to reduce the need for hospital admissions • Patients identified late have poorer outcomes of care and unnecessary hospital admissions
Switzerland	2009	Government based steering committee and expert working groups comprising of experts in palliative care	• Most people die in nursing homes yet the majority prefer to die at home • Adequate community based services enable people to stay at place of choice and avoid unnecessary hospital admissions • The patient should be supported to choose where they would like to spend their last phase of life

Through a process of thematic analysis as outlined by Braun and Clark [21] three key themes relating to palliative care and death in hospital were identified:

- Preferences for place of care and place of death outside the hospital setting
- Unnecessary or avoidable hospital admissions
- Quality of care in hospital

### Preferences for place of care and place of death outside the hospital setting

Most policies focused on achieving patient preference for end of life care, particularly in relation to setting of care. All made reference to the large numbers of people dying in public hospitals, and also cited research evidence which concludes that home is the preferred place of death for most people. The English policy referred to surveys of the general public and those with a life limiting illness to argue that “…given the opportunity and right support, most people would prefer to die at home.” (Department of Health, England p 7 [16]). They go on to say that only a small number of patients manage to die at home and most will die in an acute hospital which is not their preferred place of death.

Two policies [16, 19] referred to the fluctuation of patient preferences over the course of the illness with a tendency for patient preference to move to an in-patient setting as the illness progressed. However, these policies noted that the evidence indicated a preference for hospice rather than hospital or aged residential care. This conclusion was derived from studies [23, 24] demonstrating that patient and family satisfaction is greater in hospice compared to the hospital setting; the Irish policy stated that “Hospice inpatients reported lower levels of pain compared to their hospital counterparts in some studies. Surveys found comparatively higher levels of satisfaction with inpatient hospice care, compared to conventional, non-specialized forms of care”. (Department of Health and Children, Ireland p53) Two policies [16, 19] acknowledged the evidence that some groups, such as older people, may prefer to die in an inpatient setting to avoid being on their own or becoming a burden to family.

### Unnecessary hospital admissions

A focus on cost savings was evident throughout the reviewed policies and death in hospital was considered to be a significant cost burden to some health care systems. The Australian policy [17] stated “20 % of people die in hospices and 10 % in nursing homes. The rest die in hospitals. This results in a high cost burden for the health system and potentially a poorer quality of death”. (Commonwealth Government, Australia, p1).

Identifying and avoiding unnecessary hospital admissions was a focus for four countries to varying degrees [16–18, 20]. Reducing hospital admissions was seen as an opportunity to save hospital based spending and divert these savings to community based services in order to support patient choice with their preferred place of care which, based on the preferences for place of care and place of death studies, was presumed to be outside the hospital setting and preferably at home. Assumptions about cost savings were not evidence based nor did they consider family carer costs. The Swiss policy referred to the range of palliative care services as a ‘support network’ that would ensure that the preferred place of care and place of death is achieved whilst unnecessary hospital admissions avoided”. (Federal Office of Public Health, Swiss Confederation p5).

Policies identified a number of factors contributing to ‘unnecessary’ hospital admissions at the end of life, including a failure of community services to meet patient needs and difficulties in identifying those who would benefit from palliative care services. Timeliness of referral to palliative care services was seen as key to achieving a reduction in unnecessary hospital admissions, with the Singapore policy stating that “Patients who are identified late in the course of the illness usually have poorer outcomes of care and unnecessary hospital admissions”. (Ministry of Health, Singapore p32).

Cost savings associated with a reduction in hospital admission was considered an opportunity to increase funding for community sources [16, 17]. The English policy [16] suggests that the cost savings achieved through reducing hospital admissions could be used to improve the community based provision of palliative care stating that “It is likely, for example, that at least part of the additional costs of providing improved care in the community and in care homes will be offset by reductions in hospital admissions and length of stay”. (Department of Health, England p16). However, costs incurred by family caregivers were not considered.

### Quality of care in hospital

Research findings outlining poor quality palliative care provided in hospital settings were drawn upon throughout the reviewed policies [25, 26]. Four [16, 17, 19, 22] policies made mention of strategies to improve palliative care in the hospital setting most of which was centred around implementation of hospital based specialist palliative care teams. Other strategies such as programs to support patient preferences, choice at the end of life or facilitating discharge emphasised a need to avoid or reduce hospital admissions. All policies acknowledged the role of hospital based palliative care teams and their influence in improving care in this setting and reducing length of stay. Furthermore, the role that hospital based palliative care teams have in supporting clinicians to provide quality palliative and end of life care was highlighted in all policies. However, involvement of specialist palliative care teams were also seen as an opportunity for cost savings. For example, the Irish policy cited studies that suggest specialist hospital palliative care teams have an impact on reducing hospital inpatient bed days, increase patient time spent at home and have equal or lower costs. Furthermore, they argue that hospices (in comparison) use fewer interventional therapies and diagnostic tests whilst suggesting a further opportunity for cost savings through avoidance of hospital care.

Some policies acknowledged that the quality of palliative care across all care settings needed to improve with the English policy stating that “High quality care should be available wherever the person may be: at home, in a care home, in hospital, in a hospice or elsewhere”. (Department of Health, England p10). The hospital setting in particular was criticised as being inadequate in providing palliative care. The Irish policy cited a number of studies that demonstrated significant issues with hospital based care stating that “The care provided by hospitals was more subject to criticism than any other type of care. It found a wide range of problems with inpatient hospital care. These included an uncaring attitude, poor symptom control, and difficulty in extracting information from doctors. Poor communication was reported as the most prominent criticism…”. (Department of Health and Children, Ireland p53).

## Discussion

Some authors suggest that government policy is developed in response to a ‘social problem’ which needs fixing [27, 28]. Indeed the way in which policy is typically written implies that something needs to change, yet the ‘problem’ being addressed is often not made explicit [29]. It has been argued that identifying and interrogating ‘the problem’ underpinning policy development is important because it helps to increase our understanding of the assumptions that inform governing practices [29]. The themes identified in this review suggest a ‘problematisation’ of palliative care and death in the hospital setting. This is perhaps unsurprising as it is in line with the idea of a ‘good death’ which forms the philosophical underpinnings of palliative care and advocates for a ‘natural death’ at home surrounded by friends and family. Emulating the ideology of a ‘good death’ as currently defined may be difficult to achieve in a hospital setting.

Supporting patient preferences for place of care and place of death outside the hospital setting is a major focus across all the reviewed policies. However, the belief that place of care and place of death is an over-riding priority for patients at the end of life has been challenged. For example, a UK based study exploring the relative importance of place of death to patients with advanced cancer to achieving what they considered to be ‘a good death’ found that for some patients a home death is either unimportant or should be avoided [30]. The authors found that factors such as ‘control of pain’ and ‘not being a burden to family’ ranked higher than being able to ‘die at home’ for many participants. Preference for place of care and place of death has also been shown to vary with age, gender and ethnicity and is influenced by previous experience and concerns about being a burden [31].

Prioritising patient choice assumes a preference for individualised autonomous decision making; however this approach does not fit with all cultures. End of life decision making requires a level of complexity in relation to choice which can be difficult for some people when they are facing an uncertain and limited future [32]. Moreover, whilst the individualised approach to decision making dominates the Western model of healthcare, some non-Western cultures have been shown to demonstrate a preference for a more collective decision making approach, acknowledging that decisions made by one individual will have implications for their wider family or community [33]. In addition, in order to elicit people’s preferences for place of care and place of death there needs to be a willingness to talk openly about death and dying so that preparation can be made to fulfil their wishes at the end of life. Open acknowledgement of the imminence of death is considered to be one of the elements of a ‘good death’ [8] yet for many cultures openly talking about death may be considered detrimental to patient care which has implications for conversations regarding diagnosis and prognosis [34].

There is an implicit assumption throughout the reviewed policies that preferences regarding place of care and place of death remain stable throughout the illness trajectory. However, there is clear evidence indicating that preferences may change as a person’s circumstances evolve. Indeed, the closer to death people come, the less likely they will be to choose death at home and in fact many will choose a hospital setting [35, 36]. Moreover, while ‘home’ is commonly understood to be a fixed geographical location, research has shown that home is in fact a malleable concept [37, 38]. Indeed, studies have shown that as care needs increase the home environment changes in a way that it may no longer feel like the home patients remember. For example, an increasing need for medical equipment such as hospital beds and oxygen concentrators, along with health professionals visiting frequently, changes the nature of the home environment for some people [39].

Moreover, whilst people rarely choose the hospital as their preferred place of care at the end of life [40], the hospital may become an attractive refuge during periods of acute illness. For example, patients with palliative care needs admitted to hospital during a period of acute illness have described feeling safe and cared for while being monitored and observed by health professionals with knowledge and expertise about their illness [36]. This suggests that when care needs are changing home may feel less safe than inpatient settings.

A focus on identifying and avoiding unnecessary hospital admissions, particularly in the more recently published policies suggests that hospitalisation for those with palliative care needs is regarded as a problem [16, 17]. Whilst it might seem logical to consider a hospital admission in the context of an incurable illness to be unnecessary, particularly when the hospital is seen as an environment where life prolonging interventions take place, a Dutch study found that the most common reasons for hospital admissions in the last 3 months of life is symptom control [41]. Furthermore, half these admissions were initiated by General Practitioners, suggesting that what was occurring could not be managed in the community. In the future, the need for hospital support to initiate and monitor some palliative care interventions is likely to require more access to hospital level care [42]. However, this was not acknowledged in the policies reviewed.

Policies suggest that identifying unnecessary hospital admissions provides opportunities to save money and support patient preferences to be at home. However, there is neither an agreed definition of what an unnecessary admission is within the literature [43], nor any validated tools to identify potentially avoidable admissions in a palliative care context. Indeed differing approaches have been adopted in the literature. For example, a study by Robinson et al. [44] defined a potentially avoidable admission as one that occurred as a result of a predictable deterioration in the patient’s condition which could have been managed by community providers. In contrast, a study by Abel et al. [45] considered a hospital admission to be avoidable if the patient could have stayed at home if services as described in England’s End of Life Care Strategy were available. These differences in methodology make it difficult to support the straightforward assumption implicit in the policies reviewed that avoidable hospital admissions for those with palliative care needs can be identified; the infiltration of the ‘rescue culture’ of modern medicine also challenges the assumption they can be easily prevented [46].

Research describing poor quality of palliative care in the hospital setting was cited throughout the reviewed policies, reinforcing the argument that these are not settings where people with palliative care needs should receive care. However, findings from a recent integrative review showed that, largely due to inadequacies in study design, what is known about patient and family experiences of palliative care in a hospital setting is limited to discrete aspects of care [47]. Moreover, a study published subsequent to the review found that patients with palliative care needs experience a range of benefits associated with being in hospital that extend beyond the treatment they receive and almost all participants expressed a preference to be in hospital during a period of acute illness [44].

Overall the findings from this exploratory study suggests that Western understandings of a ‘good death’ which prioritises end of life care at home and death outside the hospital setting have informed the development of palliative care policy in countries where palliative care is integrated into mainstream health care. An emphasis on inadequate end of life care in hospitals and a focus on avoidable admissions has ‘problematised’ palliative care in the hospital setting. This policy focus has real implications for palliative care practice and ultimately patient and family experience. There is an urgent need to adopt a co-design approach to policy development to ensure that recommendations for service development meets the needs and wants of patients with palliative care needs and their family. In particular, given mounting evidence regarding patient preference for hospital admission, coupled with the increasing medicalisation of palliative care itself, future policy needs to consider what role hospitals should have at end of life, rather than assume they have none.

## Conclusion

Findings from this review suggest a ‘problematisation’ of palliative and end of life care in acute hospital settings. This approach to policy development influences service recommendations, many of which are designed to solve the ‘problem’ of people being cared for and dying in hospital. However, little is known about patient preferences for place of care during periods of acute illness or the benefits they experience from being in hospital. It has been suggested that without a better understanding of patient’s priorities and preferences at the end of life, there is a risk that the model of palliative care outlined in policy will be applied “blanket-fashion” and prove to be ineffective and inequitable [48].

### Strengths and limitations

As far as the authors are aware, this is the first study of how policy positions the acute hospital within a palliative care context. However, there are a number of limitations that need to be acknowledged. The review was limited to those policies that were available in English. The differences in health care systems may impact on the way in which policies are developed and implemented. In addition the policies were written over a decade during which palliative care has continued to evolve. Therefore, the themes identified cannot be applied across all countries nor would they necessarily be applicable in resource-poor countries. Nevertheless, the findings provide useful insights and provide a baseline for future more comprehensive reviews which is inclusive of non-English speaking countries.
